# Effectiveness of Brexpiprazole in a Patient With Bipolar Disorder and Comorbid Persistent Genital Arousal Disorder/Genito-Pelvic Dysesthesia: A Case Report

**DOI:** 10.7759/cureus.50349

**Published:** 2023-12-11

**Authors:** Yuki Hirose, Yasunori Oda, Tatsuya Kobayashi, Kazuki Okada, Masaomi Iyo

**Affiliations:** 1 Department of Psychiatry, Graduate School of Medicine, Chiba University, Chiba, JPN; 2 Department of Regulatory Science, Fujita Health University, Tokyo, JPN

**Keywords:** bipolar disorder, brexpiprazole, gpd, pgad, genito-pelvic dysesthesia, persistent genital arousal disorder

## Abstract

Although the symptoms of persistent genital arousal disorder/genito-pelvic dysesthesia (PGAD/GPD) can have negative impacts on patients' lives, it is an under-recognized clinical entity. We describe the case of a 61-year-old Japanese female who suffered simultaneously from bipolar disorder and PGAD/GPD. She developed PGAD/GPD approx. 10 years after being diagnosed with bipolar disorder. Despite 20 years of various drug treatments, her bipolar disorder and PGAD/GPD symptoms showed little improvement. She had also undergone multiple sessions of cognitive behavioral therapy (CBT) and mindfulness, nerve block, botulinum toxin injections, and laser treatment for PGAD/GPD. Her PGAD/GPD symptoms remained with no significant improvement, and her bipolar disorder symptoms had also not responded well to medication. With the administration of brexpiprazole, she achieved remission of her bipolar disorder. Her PGAD/GPD symptoms also eventually improved. When PGAD/GPD is comorbid with bipolar disorder, the improvement of bipolar disorder may also lead to relief of PGAD/GPD symptoms. This case reveals that brexpiprazole, which has a unique profile, may be effective for PGAD/GPD.

## Introduction

Persistent genital arousal disorder (PGAD) is a relatively recently described sexual disorder and an under-recognized clinical entity, characterized by symptoms of spontaneous genital arousal, which persist in the absence of sexual desire and may affect women and men [1]. The prevalence of PGAD is estimated to range from 0.6% to 3% [2], and thus a significant number of people may be affected by PGAD worldwide. PGAD was first described in 2001 by Leiblum et al. [3], and since then, the definition of PGAD has evolved, and the nomenclature of PGAD has broadened to include genito-pelvic dysesthesia (GPD). The following diagnostic criteria for PGAD/GPD were proposed by the International Society for the Study of Women's Sexual Health (ISSWSH) expert panel in 2019 [2]: persistent or recurrent, unwanted or intrusive, distressing sensations of genital arousal; duration of ≥3 months; may include other types of GPD (e.g., buzzing, tingling, burning, twitching, itch, pain); most commonly experienced in the clitoris but also in other genito-pelvic regions (e.g., mons pubis, vulva, vestibule, vagina, urethra, perineal region, bladder, and/or rectum); may include being on the verge of orgasm, experiencing uncontrollable orgasms, and/or having an excessive number of orgasms; and not associated with concomitant sexual interest, thoughts, or fantasies.

The symptoms of PGAD/GPD can, thus, have negative impacts on patients' lives. The ISSWSH panel reported that women who suffer from PGAD/GPD experience difficulty with mental health issues (e.g., depression and/or anxiety, including panic attacks and obsessive-compulsive symptoms) and significant difficulties with psychosocial adjustment. The panel further reported that the anxiety may reinforce, exacerbate, and maintain PGAD/GPD [2]. In fact, Mooney et al. observed that individuals with distressing PGAD symptoms reported significantly lower relationship and sexual satisfaction, significantly higher sexual distress, and significantly more symptoms of depression and anxiety compared to control participants [4].

Regarding the pathogenesis of PGAD, the ISSWSH expert panel concluded that there are many different specific etiologies that contribute to PGAD/GPD. They agreed that psychological factors, medical factors (e.g., pudendal neuropathy, cauda equina pathology), and pharmacological factors (e.g., selective serotonin reuptake inhibitor (SSRI) discontinuation) may all contribute to the development of PGAD/GPD [2]. There are no clinical trials examining the safety and effectiveness of treatment for PGAD/GPD, but the following therapies have been attempted: a biopsychosocial management model, including the application of anesthetizing agents to numb the area, pharmacotherapy, cognitive behavioral therapy (CBT) and mindfulness techniques, hypnotherapy, electroconvulsive therapy, pelvic floor physical therapy, botulinum toxin injections, variceal embolization, and transcutaneous electrical nerve stimulation [1].

We present the case of a female patient with simultaneous bipolar depression and comorbid PGAD/GPD. Her painful symptoms of PGAD/GPD significantly improved after she was administered the atypical antipsychotic brexpiprazole.

## Case presentation

Our patient was a 61-year-old Japanese female with bipolar disorder and PGAD/GPD. She had graduated from a national college of music and then held piano lessons in her home. She married at 23 and had one son. At the age of 39, she had been caught up in work every day and cut her sleeping hours. She gradually became euphoric and then even more overactive. After these hypomanic episodes, she developed a tendency to experience depression and anxiety. She later noted that she "couldn't think straight" and that her body suddenly stopped moving at times, and she thus began visiting a psychiatrist. She was diagnosed with bipolar disorder and began taking psychotropic medication (details unknown).

Between the ages of 42 and 49, she lived in Singapore due to her husband's work. From the age of ~43 years, she began to feel symptoms of sexual arousal, such as tingling, congestion, and throbbing in her genitalia (clitoris, vagina, and uterus). These unwanted and intrusive symptoms were not accompanied by sexual interest or desire and persisted all day at their worst. These symptoms also exacerbated the patient's anxiety. Although she saw a gynecologist in Singapore and underwent various tests, the examination revealed only mild redness in the vulva. The patient eventually told her husband, "I'd rather you kill me than continue to put me through all this pain!".

The patient tried a pain clinic, where she was diagnosed with vulvodynia (a chronic pain syndrome that affects the vulvar area without a definite organic origin). After her visit to the pain clinic, she was administered two nerve blocks at S4/S5, started using oxycodone, and then switched to fentanyl patches. These treatments helped alleviate some of her pain. At the age of 49, the patient returned to Japan and attended a nearby psychosomatic clinic. Since that time, she had been unable to leave her home due to anxiety, and her emotional highs and lows became more pronounced. Despite the wide variety of medications, including antidepressants, antipsychotics, anticonvulsants, and mood stabilizers, her symptoms did not improve by much.

In January 2022 (at the age of 59), the patient came to our hospital's Department of Anesthesiology, Pain and Palliative Care Medicine. She was subsequently examined by one of our hospital's gynecologists and was referred to our department of psychiatry in May 2022. At the time of the referral, she was regularly taking lithium carbonate 400 mg, trazodone 50 mg, tramadol hydrochloride 400 mg, and pramipexole hydrochloride hydrate 1 mg daily. She was also taking alprazolam 0.4 mg, clonazepam 0.5 mg, loxoprofen sodium hydrate 60 mg, trihexyphenidyl HCl 2 mg, and domperidone 10 mg as needed. Her blood concentration of lithium was 0.37 mEq/L. In October 2022, she received CBT and mindfulness guidance from therapists; however, this psychotherapy was discontinued due to her worsening PGAD/GPD symptoms.

In January 2023, the patient was examined at a different urology hospital and was diagnosed with PGAD/GPD. She was prescribed clomipramine for PGAD/GPD, but she discontinued it due to activation syndrome. She then received botulinum toxin injections at over 30 sites on her clitoris and vagina. She also underwent multiple sessions of IntimaLase® laser treatment on her clitoris and vagina. These treatments appeared to be temporarily successful, and her symptoms of PGAD/GPD such as tingling, congestion, throbbing, and pain were partially alleviated. However, the effects were lost after a week. When asked about her quality of life, the patient stated, in tears, "Every day from morning to night, I have congested clitoris and my uterus is twitching! I have been in pain all along!". She described feeling that she was too tired to live and wanting to die to end her agony. Since many medications had been tried by the patient in the past without success, we decided to use brexpiprazole, which she had never received, and we made no drug adjustments other than the addition of brexpiprazole.

A few days after the start of the patient's brexpiprazole medication 1 mg daily, her depressive symptoms, including depressed mood, loss of interest, and psychomotor agitation, gradually improved, and her suicidal ideation disappeared. In addition, her symptoms of PGAD/GPD improved significantly. She used to visit our department with tears in her eyes and an anguished expression on her face, and after the start of her brexpiprazole treatment, she regularly smiled during her visits. Although some symptoms remained, she was able to overlook them and live as fully as she had before she became ill. Over six months have passed since the introduction of brexpiprazole, she has voluntarily terminated her visits to a gynecologist and anesthesiologist because her PGAD/GPD symptoms had sufficiently improved.

## Discussion

We have presented the case of a patient with bipolar disorder and comorbid PGAD/GPD. Despite having been administered a wide variety of psychotropic and physical medications, the patient could not achieve remission. However, brexpiprazole ameliorated the symptoms of not only the patient's bipolar disorder but also her PGAD/GPD. To our knowledge, this is the first published report describing the effectiveness of brexpiprazole for bipolar disorder with PGAD/GPD.

Although PGAD/GPD was first reported in 2001, the mechanism underlying this disorder is still unclear. The ISSWSH expert panel stated that the presumptive pathophysiology of PGAD/GPD is sensory hyperactivity originating in any of five regions: end organs, pelvis and perineum, cauda equina, spinal cord, and brain. They further proposed a PGAD/GPD treatment algorithm, which indicates that mainly physical therapy and/or medication should be used for PGAD/GPD due to an organic origin, while psychotherapy and/or psychopharmacological treatment should be mainly used for PGAD/GPD due to a non-organic origin [2].

In fact, it has been reported that the symptoms of PGAD/GPD due to trauma and other problems responded relatively well to physical treatments [5,6]. Our patient underwent various tests, but no organic cause was observed; however, she had received many physical treatments due to the severity of her symptoms. PGAD may appear to be a form of pain disorder and/or a somatic symptom disorder, but the symptoms can be more severe than those of pain disorder and somatic symptom disorder due to the nature of the affected areas. Further investigations are needed to elucidate this issue.

It has been reported that selective serotonin reuptake inhibitors (SSRIs) and serotonin-norepinephrine reuptake inhibitors (SNRIs) may induce PGAD/GPD [7], and it is thus possible that our patient's PGAD/GPD was a side effect of SSRI or SNRI use. Unfortunately for patients, awareness of PGAD/GPD is still not widespread among psychiatrists, gynecologists, and other physicians. In addition, the nature of PGAD/GPD symptoms could make it difficult for patients to tell their doctors about painful symptoms in detail, even if they are currently suffering from these symptoms. Although our patient told us about her symptoms, physicians who provide psychopharmacological treatments should still be aware of PGAD/GPD in their daily practice.

Vulvodynia is defined as vulvar pain lasting ≥3 months without a clearly identifiable cause, and it is typically characterized by a stinging, burning, or itching sensation. Vulvodynia is a leading cause of dyspareunia in premenopausal women, causing considerable morbidity and sexual dysfunction [8]. It is not yet known whether or not PGAD/GPD is one of the subtypes of vulvodynia or a stand-alone sexual dysfunction. Considering that a multimodal approach is used to treat vulvodynia and PGAD/GPD, it appears that their common symptoms around female genitalia can be intensive and obstinate. Clinicians may, therefore, need to provide patients with PGAD/GPD or vulvodynia with comprehensive medical care, including psychopharmacological treatment and psychotherapy, as is used for patients with a pain disorder or somatic symptom disorder.

Brexpiprazole is a serotonin-dopamine activity modulator (SDAM) that acts as a partial agonist at dopamine D2/3 and 5-HT1A receptors and as an antagonist at 5-HT2A, 5-HT2C, 5-HT7, and noradrenaline alpha1B/2C receptors [9,10]. Brexpiprazole is, thus, used for patients with schizophrenia and as adjunctive therapy with antidepressants for individuals with major depressive disorder. Given that both the activation of 5-HT1A and the inhibition of 5-HT7 have antidepressant- and anxiolytic-like effects [11], brexpiprazole could be also effective for bipolar depression, similar to lurasidone. In fact, it has been reported that brexpiprazole ameliorated the symptoms of bipolar depression [12]. Brexpiprazole is, thus, being studied for the treatment of bipolar depression.

Considering that the stimulation of 5-HT7 on peripheral nociceptors increases pain perception [13], it is possible that the inhibition of 5-HT-7 may decrease pain. It is unclear whether the improvement in mood leads to the symptomatic relief of PGAD/GPD or whether it has a direct effect on PGAD/GPD, but these unique properties of brexpiprazole might have contributed to the relief of our present patient's pain as well as her depression.

## Conclusions

In conclusion, we administered brexpiprazole to a patient with bipolar disorder and comorbid PGAD/GPD. Although her symptoms of bipolar disorder and PGAD/GPD had shown little improvement despite a wide variety of treatments, the patient was able to achieve remissions of both her bipolar disorder and her PGAD/GPD after the introduction of brexpiprazole. It is possible that the unique profile of brexpiprazole as an SDAM is responsible for improvements in not only bipolar disorder but also PGAD/GPD. In addition, physicians should be aware of PGAD/GPD in their daily practice. Further clinical research is necessary to clarify the effects of brexpiprazole in these disorders.
